# Incorporation of heparin/BMP2 complex on GOCS-modified magnesium alloy to synergistically improve corrosion resistance, anticoagulation, and osteogenesis

**DOI:** 10.1007/s10856-021-06497-8

**Published:** 2021-03-06

**Authors:** Yuebin Lin, Ya Yang, Yongjuan Zhao, Fan Gao, Xin Guo, Minhui Yang, Qingxiang Hong, Zhongmei Yang, Juan Dai, Changjiang Pan

**Affiliations:** 1 Faculty of Mechanical and Material Engineering, Huaiyin Institute of Technology, Huai’an, 223003 China; 2 The Affiliated Huai’an Hospital of Xuzhou Medical University, Huai’an, 223003 China

## Abstract

The in vivo fast degradation and poor biocompatibility are two major challenges of the magnesium alloys in the field of artificial bone materials. In this study, graphene oxide (GO) was first functionalized by chitosan (GOCS) and then immobilized on the magnesium alloy surface, finally the complex of heparin and bone morphogenetic protein 2 was incorporated on the modified surface to synergistically improve the corrosion resistance, anticoagulation, and osteogenesis. Apart from an excellent hydrophilicity after the surface modification, a sustained heparin and BMP2 release over 14 days was achieved. The corrosion resistance of the modified magnesium alloy was significantly better than that of the control according to the results of electrochemical tests. Moreover, the corrosion rate was also significantly reduced in contrast to the control. The modified magnesium alloy not only had excellent anticoagulation, but also can significantly promote osteoblast adhesion and proliferation, upregulate the expression of alkaline phosphatase and osteocalcin, and enhance mineralization. Therefore, the method of the present study can be used to simultaneously improve the corrosion resistance and biocompatibility of the magnesium alloys targeted for the orthopedic applications.

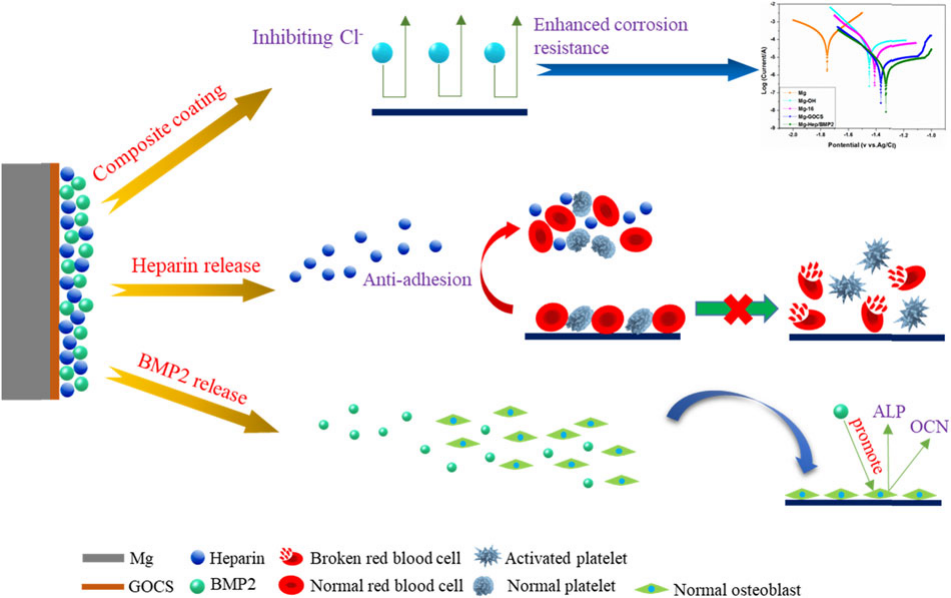

## Highlights

- The GOCS@Hep/BMP2 bioactive coating was created on the magnesium surface.
- The coated magnesium alloy had good wettability and corrosion resistance.
- The coating can improve anticoagulation and osteogenesis of magnesium alloy.

## Introduction

The tendency toward the biodegradable implant is attracting more and more attention due to the increase of orthopedic traumas [1, 2]. In this aspect, magnesium and its alloys have been considered the ideal candidate as a permissible implant material to support the structure temporarily, and safely degrade in a body [3]. First, the density and mechanical properties of the magnesium alloy are close to human bone so that it can effectively avoid the stress shielding effect when used as a bone implant material [4]. Second, magnesium can be degraded in the physiological environments in the form of Mg^2+^, and the degradation products can be excreted out of the body or absorbed by the human body. Consequently, as a promising biodegradable metal, it can not only avoid being removed by the second operation, but also reduce the risk of physical pain of patients and reinfection [5].

Although magnesium alloy has no obvious toxicity to human body and can even promote the healing of bone tissue in some cases [6–8], its rapid degradation in the physiological conditions usually exceeds the threshold that the human physiological environment can withstand. A large number of hydrogen gas and magnesium ions, as well as rapidly rising pH value, can not only destroy the mechanical integrity of the loading–bearing implant, but also break the in vivo physiological balance, leading to serious inflammatory and toxic reactions at the interface between the implant and human tissues, finally resulting in the implantation failure. These key problems regarding the application and safety of the magnesium alloy must be mitigated by the effective strategies before the application.

Surface modification represents one of the key and effective methods to enhance the corrosion resistance and biocompatibility because the corrosion behaviors and biocompatibility of the magnesium alloys are closely related to their surface properties [9]. At present, many surface modification methods have been employed to control the surface anticorrosion and bioactivities of the magnesium alloys [10–12]. By forming a chemical conversion layer or coating on the magnesium surface to isolate the matrix from the corrosive environment, the corrosion resistance can be significantly improved, which could reduce a series of adverse physiological reactions caused by the rapid degradation, and thus improve the biocompatibility to a certain extent. However, these methods have limited improvement on the surface properties and functions of the magnesium alloys because of the thinner surface-modified layer, small number of biomolecules, and easy deactivation of bioactive substances. Although the polymer or Ca-P coating on the magnesium alloy surface has been extensively investigated and they can significantly improve the corrosion resistance, both bioactivities and corrosion resistance in the in vivo environments are inferior to the clinic requirements. On the other side, studies have shown that the poor anticoagulation of the bone implant would induce platelet adhesion and activation, resulting in thrombus and poor blood flow at the implantation site, even local tissue necrosis [13]. Therefore, magnesium alloys used for the bone implant materials not only need to control their degradation behavior in vivo, but also should be endowed with good osteogenic and anticoagulant properties.

Graphene oxide (GO), a 2D carbon nanomaterial, has been widely investigated in the field of biomaterials and tissue engineering because of its huge specific surface area, high mechanical strength, and good biocompatibility [14, 15]. GO has rich carboxyl groups showing negative-charged characteristic in electrolyte solution, which can prevent the adsorption of anions and thus improve the corrosion resistance and the biocompatibility of the magnesium alloys [16–18]. At the same time, the aromatic ring structure of GO can enhance the extracellular matrix (ECM) protein adsorption through non-covalent interaction, which can improve cell proliferation and differentiation [19, 20]. In addition, the rich hydroxyl, carboxyl, and epoxy groups of GO can be linked with other bioactive substances to achieve multi-functionalization. Moreover, its huge specific surface area also provides an excellent platform for loading bioactive molecules and drugs to enhance the bioactivities [21]. Chitosan has many bioactivities including good biocompatibility and antibacterial activity, and it is widely used in the biomaterials and tissue engineering. Consequently, the bioactivities of GO can be further improved by chitosan. Chitosan has also been demonstrated as the ability to enhance corrosion of the magnesium alloy [22]. Therefore, the immobilization of chitosan-functionalized GO (GOCS) on the magnesium alloy surface can not only improve the corrosion resistance and biocompatibility, but also provide an excellent platform for loading bioactive substances.

Heparin has been extensively used for the surface modification of biomaterials to improve the blood compatibility. Heparin has excellent binding affinities to various factors, such as vascular endothelial growth factor (VEGF) and BMP2 [23], and its strong negative-charged characteristics endow its good abilities to bind with the positively charged biomolecules [24]. BMP2 can promote osteoblast proliferation and differentiation [25, 26]. Meanwhile, BMP2 can bind to heparin due to its positively charged characteristic. Therefore, due to the positive-charged characteristic and huge specific area of GOCS, the complex of heparin and BMP2 can be further loaded on the surface of GOCS-modified magnesium alloy to improve the blood compatibility and promote osteoblast growth through the sustained release of heparin and BMP2.

In view of the bioactivities of GO, chitosan, heparin, and BMP2, a strategy for the surface modification of the magnesium alloy is proposed in the present study. First, GOCS was covalently immobilized on the magnesium alloy surface, which was modified by self-assembly of 16-phosphonyl-hexadecanoic acid. Subsequently, the complex of heparin and BMP2 was loaded on the modified surface to enhance the anticoagulation and osteogenesis through the sustained release of heparin and BMP2. The advantage of this method is that a multifunctional surface can be achieved, which can not only improve the corrosion resistance of the magnesium alloy, but also improve its blood compatibility and osteogenesis.

## Materials and methods

### Incorporation of heparin/BMP2 on GOCS-modified magnesium alloy surface

1 mg/mL GO suspension (XFNANO Materials Tech Co., Ltd, Nanjing, China) and 5 mg/mL chitosan (0.2% acetic acid, pH 5.0) solution were thoroughly mixed 12 h with a ratio of 1:5 (volume ratio), and then 10 mM 1-(3-dimethylaminopropyl)-3-ethylcarbodiimide/N-hydroxysuccinimide (EDC/NHS, volume ratio 3:1) was added under stirring at 800 rpm. After 12 h reaction at room temperature, the mixture solution was transferred into a dialysis bag (MW:3500) to dialyze 5 days in deionized water, the remaining solution was finally centrifuged and dried to obtain GOCS.

AZ31B magnesium alloy plates were polished by SiC paper up to 2000 grits. After cleaning and drying, the plates were immersed in 3 M NaOH solution for 24 h at 75 °C, and the obtained samples were recorded as Mg-OH. Mg-OH was immersed in 5 mM 16-phosphonyl-hexadecanoic acid solution (in anhydrous ethanol) for 24 h, then taken out and treated at 120 °C for 12 h, the sample was recorded as Mg-16. Mg-16 was activated in EDC/NHS (10 mM) for 1 h, then reacted with GOCS solution (6 mg/mL) for 2 h to obtain GOCS-modified sample (Mg-GOCS). Finally, 2 mg/mL heparin solution and 100 ng/mL BMP2 solution were fully mixed by 1:1 (vol%), and then dropped and fully covered on Mg-GOCS surface for adsorbing 6 h to obtain the final sample (Mg-Hep/BMP2).

### Surface characterization

Attenuated total reflection Fourier transform infrared spectroscopy (ATR-FTIR, TENSOR27, Bruker of Germany) was utilized to examine the surface chemical groups. The scanning range was 4000–650 cm^−1^. Mg and Mg-Hep/BMP2 were analyzed by Raman spectroscopy (Renishaw, UK, RM 2000) to investigate the GO structure on the surface. The surface morphologies and element composition were investigated by scanning electron microscopy (SEM, FEI Quata 250, USA) and X-ray energy dispersive spectroscopy (EDS, IMA X-Max 20, Britain), respectively. The water contact angle was measured by a DSA25 contact angle meter (Krüss GmbH, Germany) in order to characterize the surface hydrophilicity. Three parallel samples were measured and the values were expressed as mean ± standard deviation.

### Release profiles of heparin and BMP2

The Mg-Hep/BMP2 sample was immersed in 2 mL Hanks simulated body fluid (SBF) solution. At each predetermined time, 100 μL solution was taken out and the medium was supplemented with 100 μl fresh SBF. The release amount of BMP2 was measured by BMP2 enzyme-linked immunosorbent assay (ELISA) kit [27]. Briefly, 100 μL standard solution and 100 μL sample solution were, respectively, added into the test plate. After cultivated 90 min at 37 °C, 100 μL of Biotinylated anti-mouse BMP2 antibody was added to incubate 60 min, followed by addition of 100 μL Avidin-Biotin-Peroxidase Complex solution. Finally, 90 μL chromogenic agent was added to incubate 20 min, and the terminating solution was finally added to stop the reaction. The absorbance at 450 nm was measured and the concentration of BMP2 was calculated according to the standard curve.

The concentration of heparin was measured by azure A staining method described in our previous work [28]. In brief, 100 μL of the sample solution was added to the 96-well culture plate, and then 100 μL of azure A solution was added. Subsequently, the absorbance at 505 nm was measured. The heparin concentration was calculated based on the standard curve and the release curve was plotted.

### Electrochemical and biodegradable behaviors

#### Potentiodynamic polarization curves and electrochemical impedance spectroscopy (EIS)

The potentiodynamic polarization curves and EIS of the different samples were measured on a CHI660D electrochemical workstation using the standard three-electrode system including a Pt plate as the auxiliary electrode, an Ag/AgCl electrode with 3 M KCl as the reference electrode, and the sample as the working electrode. The test was conducted in Hanks SBF. The sample was sealed with silicone rubber with copper wire as the conductor. The exposed area was about 1 cm^2^. Before testing, the sample was soaked in SBF until the open circuit potential was stable. The scanning speed was 1 mV/s. The corrosion current density was fitted by Tafel extrapolation method, and the annual corrosion depth was calculated according to the following formula [29]:

1 $${d} = 3.28 \times 10^{ - 3}\left( {{M}/{n}\rho } \right){I}_{{\mathrm{corr}}}$$

where *d* represents annual corrosion depth (mm/year), *M* is the molar mass of Mg (24 g/mol), *n* is the valence of Mg (*n* = 2), *ρ* is the density of magnesium (1.74 g/cm^3^), and *I*_corr_ is the corrosion current density (μA/cm^2^).

The EIS was also done on the Chi660D electrochemical workstation by scanning from 10^5^ to 0.1 Hz at open circuit potential with a sinusoidal alternating current amplitude of 10 mV.

#### pH changes and Mg^2+^ release

The magnesium alloy sealed with silicone rubber was immersed in 20 mL SBF solution, and the medium was refreshed every 2 days. The pH value of SBF was measured by pH meter at the predetermined time (1–7, 10, and 14 days), three parallel samples were measured and then the values were averaged.

For Mg^2+^ release, the sealed sample was immersed in 5 mL SBF solution, and then 200 μL solution was taken out at the different times (1–7, 10, and 14 days) and supplemented with 200 μL fresh SBF. The Mg^2+^ release concentration was measured by inductively coupled plasma atomic emission spectrometer (ICP-AES, Optima 7000 DV). Three parallel samples were measured and then the average value was calculated.

### Anticoagulation

#### Platelet adhesion

The anticoagulant whole blood of a healthy volunteer was centrifuged 15 min at 1500 r/min to obtain platelet-rich plasma (PRP). 200 μL PRP was fully covered on the surface to incubate 2 h at 37 °C. After washing with PBS, the attached platelets were fixed by 2.5% glutaraldehyde solution at 4 °C. The sample was successively dehydrated with 50, 70, 90, 100% ethanol solutions. After dried and sputtered a gold layer, the platelets were observed by SEM (FEI Quata 250, USA). Ten SEM images with small magnifications (×500) were randomly selected to count the platelets, and the values were averaged and expressed as platelets per unit area.

#### cGMP assay

The cGMP was measured by ELISA to characterize the platelet activation [30]. The samples were incubated with PRP for 1 h at 37 °C, and then 100 μL Triton-X was introduced. After sonication, the mixture was centrifuged at 3000 rpm, and the supernatant was analyzed according to the instruction of the cGMP ELISA kit.

#### Hemolysis

The red blood cells were obtained by centrifuging the anticoagulant whole blood of a healthy volunteer. The hemolysis rate was obtained according to our previous method [31]. The normal saline was used to prepare 2% red blood cell suspension. 2 mL suspension was incubated 3 h with the sample at 37 °C. Subsequently, 1 mL solution was taken out and centrifuged 5 min at 3000 r/min, 200 μL supernatant was used to measure the absorbance at 450 nm by a micro-plate reader (Bio-Tek, Eons). The 2% red blood cell solution by normal saline was used as the negative control, and the 2% red blood cell by the distilled water was used as the positive control. The following formula is used for calculating the hemolysis rate:

2 $${\mathrm{Hemolysis}}\,\left( \% \right) = \left( {{A}_{{\mathrm{sample}}} - {A}_{{\mathrm{negative}}}} \right)/\left( {{A}_{{\mathrm{positive}}} - {A}_{{\mathrm{negative}}}} \right) \times 100\%$$

where *A*_sample_ represents the OD value of sample, *A*_negative_ and *A*_positive_ are the OD values of negative control and positive control, respectively.

### Osteoblast behaviors

#### Cell adhesion

The samples were sterilized by ultraviolet for 12 h, and then 0.5 mL osteoblast (MC3T3-E1, Nanjing Cobioer Bio-Technology Co., Ltd) suspension (passage 4, 5 × 10^4^ cells/mL) and 1.5 mL culture medium were added for culturing for 1 and 3 days at 37 °C in a humidified incubator with 5% CO_2_, respectively. After washing with PBS, the cells were fixed by 2.5% glutaraldehyde at 4 °C. The attached cells were successively stained by rhodamine and 4, 6-diamidino-2-phenylindole (Sigma-Aldrich, Shanghai), and finally the cells were observed by a fluorescent microscopy (Carl Zeiss, Inverted A2 Imager).

#### Cell counting kit-8 (CCK-8) assay

The osteoblasts were seeded on the sample surface and cultured as mentioned above. After culturing for 1 and 3 days, respectively, the sample was transferred into a new culture plate and then 0.5 ml of 10% CCK-8 solution was added for culturing for 3.5 h. Finally, 100 μL medium was taken out to measure the absorbance at 450 nm. Three parallel samples were measured and the values were averaged.

#### Alkaline phosphatase (ALP) and osteocalcin (OCN) expression

ALP and OCN expression of osteoblasts was evaluated by ELISA. First, 0.5 ml osteoblasts with a concentration of 5 × 10^4^ cells/ml were inoculated on the sample surface for 1 and 3 days, respectively. The supernatant was analyzed according to the instruction of the ALP and OCN ELISA kit. The concentrations of ALP and OCN were calculated according to the standard curve.

#### Mineralization

The osteoblast mineralization was determined by Alizarin red S staining method described elsewhere [32]. First, the cells were seeded on the sample surface for culturing for 7 and 21 days, respectively. The attached cells were fixed by 2.5% glutaraldehyde solution for 30 min. After cleaning, 40 mM Alizarin red S (pH 4.1) solution was added on the surface for incubating 30 min. The cells were scraped off the surface with a cell scraper and transferred to 10% acetic acid solution, then the solution was heated at 85 °C for 10 min and centrifuged. Finally, the supernatant was neutralized with 10% ammonia hydroxide, and the absorbance was measured at 405 nm.

### Statistical analysis

SPSS software was used to analyzed the results of water contact angle, anticoagulation (including platelet number, platelet activation, and hemolysis rate), osteoblast proliferation (CCK-8), and function (ALP, OCN, and mineralization) using one-way ANOVA, and *p* < 0.05 is considered to be statistically significant. All tests were done three times with three parallel samples.

## Results and discussion

### Sample characterization

**Fig. 1:**
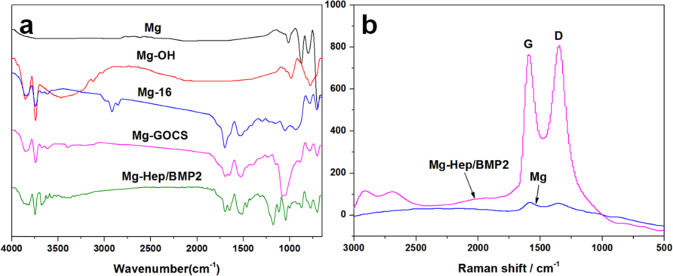
**a** ATR-FTIR curves of the different samples, the measurements were performed at room temperature with the scanning scope of 4000–650 cm^−1^; **b** Raman spectra of Mg and Mg-Hep/BMP2, the sharp and clear G and D peaks of GO were observed on Mg-Hep/BMP2

The ATR-FTIR curves of the different samples are shown in Fig. 1a. No obvious infrared absorption can be detected on Mg surface, indicating that the pristine Mg surface had no chemical groups. In contrast, an obvious infrared absorption peak at 3700 cm^−1^ can be detected on Mg-OH, which was associated with the infrared absorption of hydroxyl stretching vibration of Mg(OH)_2_ [33], demonstrating that alkali heat treatment can produce a large number of hydroxyl groups in the form of Mg(OH)_2_ on the magnesium surface. The 16-phosphonyl-hexadecanoic acid can be further immobilized on Mg-OH surface by the dehydration reaction between phosphonyl and hydroxyl. Therefore, after the immobilization of 16-phosphonyl-hexadecanoic acid, the infrared absorption peaks of methyl and methylene can be observed at 2850 and 2920 cm^−1^, respectively, and the C=O infrared absorption of carboxyl also appeared at 1720 cm^−1^, indicating that 16-phosphonyl-hexadecanoic acid was successfully introduced on the surface. The surface carboxyl group can covalently react with the amine group of GOCS in the form of amide so that GOCS can be grafted on the surface. Therefore, after the immobilization of GOCS, the infrared absorption of carbonyl group in amide group can be detected at 1680 cm^−1^, concurrently the bending vibration absorption of N–H and C–N can be found at 1530 and 1400 cm^−1^, respectively. The strong and sharp peak at 1100 cm^−1^ on Mg-GOCS can be attributed to the adsorption of C–O. Heparin and bone morphogenetic protein 2 (Hep/BMP2) displayed negative-charged characteristic. After loading on the positively charged GOCS-modified surface, the peaks at 1210 and 780 cm^−1^ on Mg-Hep/BMP2 can be attributed to S=O and S–O stretching vibrations, respectively, which indicated the successful introduction of heparin. Due to the electrostatic interaction between GOCS and heparin, the C–O peak shifted from 1100 to 1035 cm^−1^ [34]. The peak at 1700 cm^−1^ belonged to the vibrational absorption of carbonyl group. In order to further investigate the surface chemical structure, the Raman spectra of Mg and Mg-Hep/BMP2 were measured, and the results are shown in Fig. 1b. It can be seen that there was no obvious Raman absorption on Mg surface. Two sharp peaks at 1590 and 1350 cm^−1^ on Mg-Hep/BMP2 can be attributed to the G peak and D peak of GO [35], indicating that GO has been successfully introduced on the magnesium surface.

**Fig. 2:**
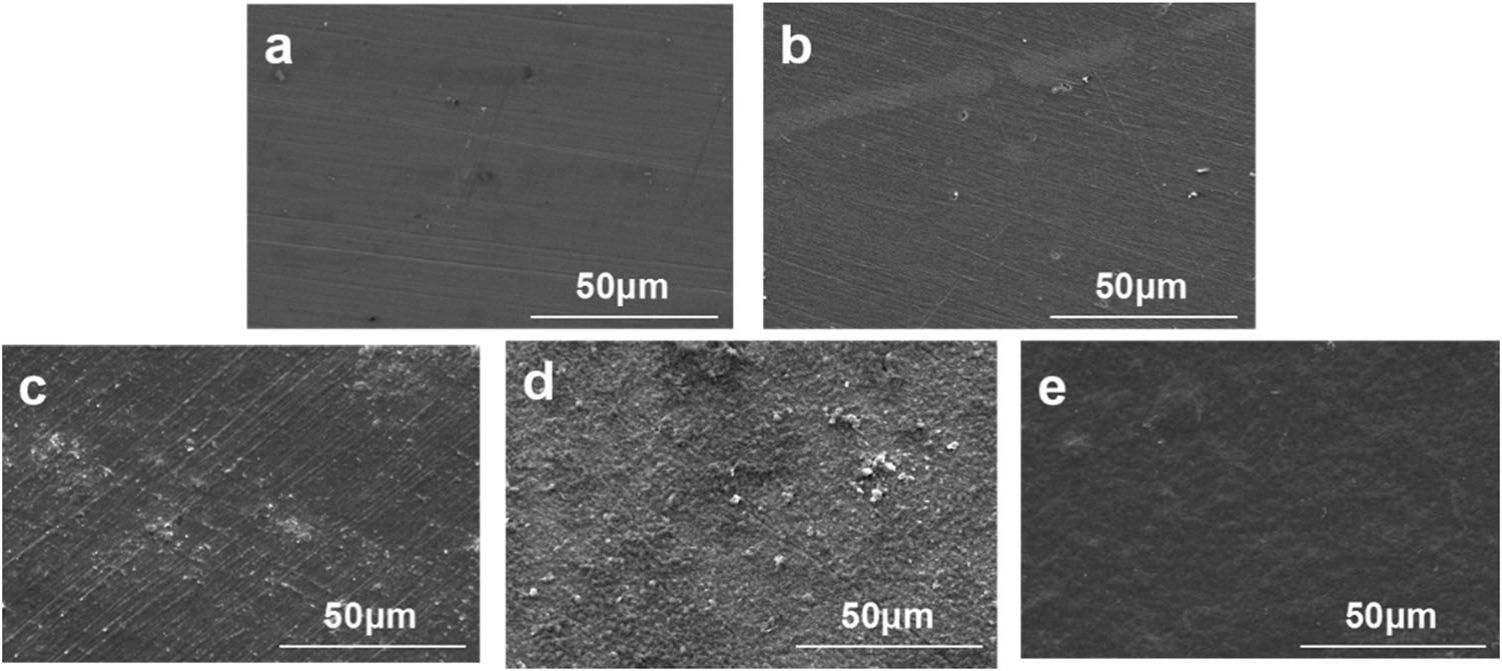
Representative SEM images elucidating the morphologies of Mg (**a**), Mg-OH (**b**), Mg-16 (**c**), Mg-GOCS (**d**), and Mg-Hep/BMP2 (**e**)

The surface morphologies and element compositions of the pristine and modified magnesium alloys were further characterized by SEM and EDS, respectively, and the results are shown in Fig. 2 and Table 1. No special morphologies on Mg surface were observed besides a few scratches caused by polishing, and the surface elements were mainly composed of Mg and O, indicating that the magnesium alloy can be oxidized in air. Alkali heat treatment can produce Mg(OH)_2_ layer on the surface, so the amount of Mg on the surface decreased concurrently with the increase of oxygen, however, there was no obvious difference for micro-morphologies between Mg and Mg-OH. The occurrence of C and P elements on Mg-16 surface demonstrated that 16-phosphonyl-hexadecanoic acid was successfully immobilized on the magnesium alloy surface, which can form an organic molecular layer covering the surface to enhance the corrosion resistance to some degree. As shown in Fig. 2d, the immobilized GOCS showed a typical granular morphology of chitosan [36], and the coating was continuous and intact, which can provide the isolation layer to enhance the corrosion resistance [37]. The C content on Mg-GOCS surface increased to 35.29% due to the introduction of GO and chitosan, and the appearance of nitrogen mainly came from chitosan, indicating that GOCS was successfully immobilized on the magnesium alloy surface. After loading Hep/BMP2, 7.29% S element can be detected, suggesting that heparin was successfully introduced. The granular structure of chitosan disappeared and a uniform and smooth coating can be observed, the content of Mg was also reduced to 2.74%, indicating that the surface coating was thicker and can completely cover the magnesium alloy surface.

**Table 1:** Elemental compositions of the different surfaces characterized by EDS

Samples	Atomic concentration (at. %)							
	Mg	O	Al	C	P	N	S	Na
Mg	87.5	9.9	2.6	–	–	–	–	–
Mg-OH	78.8	20.2	1.0	–	–	–	–	–
Mg-16	54.3	23.34	–	21.79	0.57	–	–	–
Mg-GOCS	23.36	34.24	–	35.29	–	7.11	–	–
Mg-Hep/BMP2	2.74	38.22	–	41.47	–	9.48	7.29	0.80

**Fig. 3:**
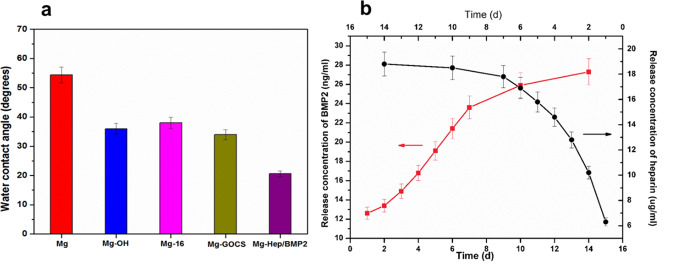
**a** Water contact angles of the different samples, the data are statistically significant (*p* < 0.05) as compared to the blank magnesium; **b** heparin and BMP2 release profiles of Mg-Hep/BMP2 for 14 days

Human body contains a plenty of water, the biomaterials will be in contact with the in vivo environment after the implantation, and therefore the surface wettability plays an important role on the biocompatibility. In the present study, the surface wettability of the different magnesium alloys was characterized by water contact angle, and the results are shown in Fig. 3a. The water contact angle of the untreated magnesium alloy was 54°, and the value is close to the results shown in literature [38], displaying the relative hydrophobicity in contrast to other samples. As shown in ATR-FTIR, alkali heat treatment can introduce a large amount of hydroxyl groups on the surface, which can significantly contribute to enhancement of the hydrophilicity, therefore the water contact angle of Mg-OH was reduced to 36°. Although the immobilization of 16-phosphonyl-hexadecanoic acid consumed the hydrophilic hydroxyl groups, another hydrophilic carboxyl groups can be introduced on the surface, therefore the water contact angle of Mg-16 had little change in contrast to Mg-OH. Although chitosan has better hydrophilicity, GO contains the aromatic hydrophobic structures, consequently, as compared to Mg and Mg-OH, the change of water contact angle was not obvious after GOCS was grafted onto magnesium alloy surface. The surface of heparin contains a lot of hydrophilic sulfonic and hydroxyl groups, therefore, the water contact angle decreased significantly after Hep/BMP2 was loaded on the surface.

GOCS with the positive charges can adsorb the negative-charged heparin/BMP2 complex, moreover, GO can be used to load heparin and BMP2 through non-covalent forces such as hydrophobic force and hydrogen bond. Its large specific surface area can effectively increase the loading amount of the bioactive substances. The release behaviors of BMP2 and heparin are shown in Fig. 3b. It can be seen that both BMP2 and heparin had an obvious burst release behavior of 1 day, and the concentration of BMP2 and heparin was 12.6 ng/mL and 6.3 μg/mL, respectively. It was considered that the burst release was related to the low binding capacity of the bioactive substances existing on the outermost layer of the surface. From the 1st day to the 7th day, the release concentration increased almost linearly with the function of time, and the daily release concentration was 1.83 ng/mL (BMP2) and 1.92 μg/mL (heparin), respectively, which was significantly lower than that of the 1st day. Although the release rate slowed down obviously, the release rate was stable. The release rate of BMP2 and heparin was further decreased from the 7th day to the 14th day, and the cumulative release concentrations of BMP2 and heparin at 14 days were 27.3 ng/mL and 18.8 μg/mL, respectively, indicating that BMP2 and heparin release could release at least 14 days from the surface. The sustained release was beneficial to promote the differentiation and proliferation of osteoblasts and can endow the materials with good anticoagulant properties.

### Electrochemical and biodegradable behaviors

**Fig. 4:**
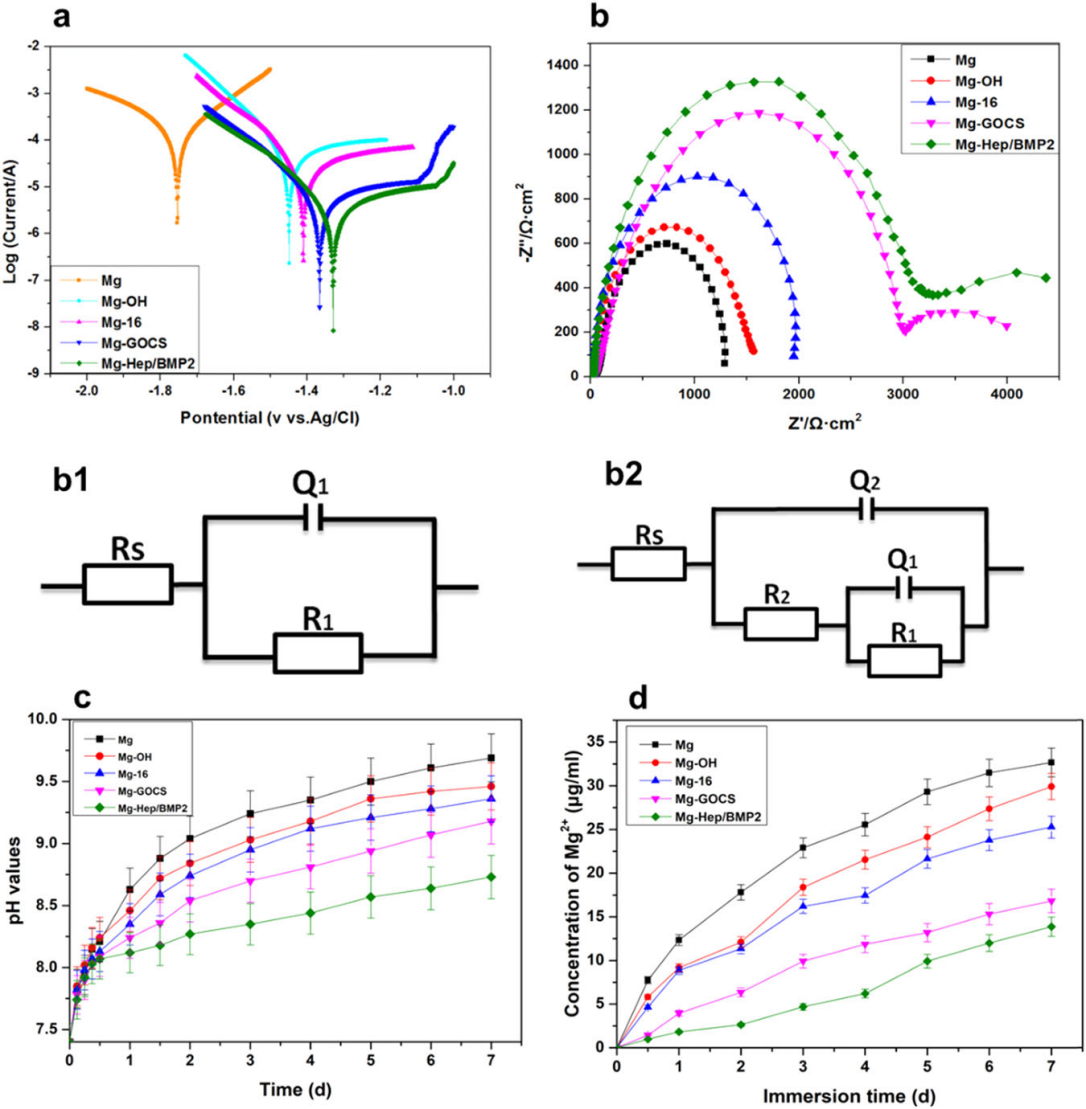
**a** Potentiodynamic polarization curves of the different samples; **b** Nyquist plots of the different samples; scatter: original values; line: calculative values; b1 equivalent circuits of Mg, Mg-OH and Mg-16; b2 equivalent circuits of Mg-GOCS and Mg-Hep/BMP2. **c** pH values and Mg^2+^ release profile (**d**) of the incubation medium solutions for the different samples which immersed in SBF for 7 days

The potentiodynamic polarization test is first done to evaluate the corrosion behaviors of the different samples, and the corrosion potential and corrosion current density were determined by Tafel method. The annual corrosion depth was calculated according to corrosion current density and Formula (1), and the results are shown in Fig. 4a and Table 2. In general, the more positive corrosion potential and the lower corrosion current indicate better corrosion resistance [39]. The corrosion potential of Mg (−1.758 V) was the lowest among all samples, indicating that Mg had poor corrosion resistance. The corrosion current density and annual corrosion depth were 6.593 × 10^−5^ A/cm^−2^ and 1.49 mm/year, respectively, representing the fastest corrosion rate in contrast to other samples. The corrosion potential of Mg-OH was positively shifted to −1.449 V, indicating that the corrosion resistance was improved due to the formation of Mg(OH)_2_ layer, therefore the corrosion current density and the annual corrosion depth of Mg-OH were reduced to 2.567 × 10^−5^ A/cm^−2^ and 0.58 mm/year, respectively. However, Mg(OH)_2_ layer is not stable and can react with Cl^−^ of the physiological environment to transform it into easily soluble MgCl_2_ [40]. In addition, although Mg-OH sample had better hydrophilicity, it needed to be further modified because of the limited surface bioactivities. The self-assembly of 16-phosphonyl-hexadecanoic acid had the phosphating effect and covering effect on the surface, consequently, the corrosion potential of Mg-16 sample increased, while the corrosion current density and annual corrosion depth decreased to some degree. As shown in SEM images (Fig. 2), the immobilization of GOCS can form a dense layer on the magnesium alloy surface, which can effectively isolate the corrosion medium from the matrix, leading to the improvement of the corrosion resistance. At the same time, the corrosion of the magnesium alloy could lead to local alkalization due to the increase of pH value, but chitosan is not easy to dissolve in alkaline environment, which also contributed to improve the stability of GOCS coating, therefore the corrosion potential of Mg-GOCS increased obviously (−1.366 V), while corrosion current density (7.682 × 10^−6^A/cm^−2^) and annual corrosion depth (0.17 mm/year) were further reduced. After loading Hep/BMP2 on Mg-GOCS, the corrosion potential slightly increased to −1.328 V, and the corrosion current density and annual corrosion depth decreased to 5.132 × 10^−6^A/cm^−2^ and 0.12 mm/year, respectively, indicating that the corrosion resistance was further improved. It may be due to the fact that the negative-charged heparin can prevent the adsorption of anions. At the same time, it can also be seen from Fig. 2 that after loading heparin and BMP2, the coating became more uniform, complete, and compact, which could be helpful to improve the corrosion resistance.

**Table 2:** Corrosion potential, corrosion current density, and annual corrosion depth of the different samples

Samples	*E*_corr_/*V*	*I*_corr_/*A* cm^−2^	d/(mm/y)
Mg	−1.758	6.593 × 10^−5^	1.49
Mg-OH	−1.449	2.567 × 10^−5^	0.58
Mg-16	−1.410	9.856 × 10^−6^	0.22
Mg-GOCS	−1.366	7.682 × 10^−6^	0.17
Mg-Hep/BMP2	−1.328	5.132 × 10^−6^	0.12

The electrochemical corrosion behaviors of the different samples were further examined by EIS, and the Nyquist curves are shown in Fig. 4b. In order to quantitatively describe the EIS response, the corresponding electrochemical equivalent circuits are constructed and shown in Fig. 4b1 (for Mg, Mg-OH, and Mg-16) and Fig. 4b2 (for Mg-GOCS and Mg-Hep/BMP2). The EIS curves were fitted with the corresponding electrochemical equivalent circuits, and the fitting parameters are shown in Table 3. It can be seen from Fig. 4b that the shape of the Nyquist diagrams of all samples was similar in the high-frequency regions, which was a capacitive loop in the shape of a semicircle attributed to charge transfer, however, the size of the semicircle was different. As shown in Table 3, it was obvious that there was no significant difference in the values of the solution resistance (*R*_*s*_) for all samples. Mg, Mg-OH, and Mg-16 had only one high-frequency capacitive ring, which can be attributed to charge transfer effect. The increasing size of the capacitive ring after the surface modification indicated that the charge transfer through the double electric layer became more difficult, leading to the increase of the charge transfer resistance (*R*_1_). After GOCS was immobilized on the magnesium alloy surface, apart from the increasing size of the high-frequency capacitance ring, the low-frequency capacitance ring can be detected. The low-frequency capacitance ring is related to the mass transfer and diffusion of Mg^2+^ caused by corrosion dissolution [41, 42]. The larger size of the low-frequency capacitance ring generally indicates that it is more difficult for metal ions to pass through the surface coating. The occurrence of low-frequency capacitance ring on Mg-GOCS suggested that GOCS layer can prevent the diffusion of Mg^2+^, resulting in the improvement of the corrosion resistance. Loading heparin and BMP2 on Mg-GOCS resulted in the thicker and more uniform coating, therefore the high-frequency capacitance ring of Mg-Hep/BMP2 was further increased and it was the largest one among all samples. As compared to Mg-GOCS, the coating resistance (*R*_2_) of Mg-Hep/BMP2 was significantly higher than that of Mg-GOCS, indicating that its corrosion resistance was the best. From the EIS fitting parameters shown in Table 3, it can be seen that the polarization impedance of Mg was only 1467 Ω cm^2^, and the value was the lowest among all samples, showing the poor corrosion resistance. Alkali heat treatment and surface immobilization of 16-phosphonyl-hexadecanoic acid can effectively improve the corrosion resistance, so the polarization impedance increased. After the bioactive coating was prepared, the polarization impedance further increased, and the impedance value of Mg-Hep/BMP2 reached 3775 Ω cm^2^, demonstrating that the coating can significantly improve the corrosion resistance. It was considered that the coating was stable in alkaline environment and can effectively prevent the adsorption of corrosive anions.

**Table 3:** The EIS fitting parameters of the different samples

Samples	*R*_*s*_/(Ω cm^2^)	*Q*_1_/(µF cm^−2^)	*n*_1_	*R*_1_/(Ω cm^2^)	*Q*_2_/(µF cm^−2^)	*n*_2_	*R*_2_/(Ω cm^2^)	*R*_*p*_/(Ω cm^2^)
Mg	38.24	3.529 × 10^−6^	0.8964	1467	–	–	–	1467
Mg-OH	37.95	3.338 × 10^−6^	0.8867	1902	–	–	–	1902
Mg-16	38.16	5.463 × 10^−7^	0.9024	2386	–	–	–	2386
Mg-GOCS	38.22	5.008 × 10^−7^	0.8631	3204	7.581 × 10^−6^	0.9206	54	3258
Mg-Hep/BMP2	38.31	3.542 × 10^−7^	0.8752	3654	4.526 × 10^−6^	0.8867	121	3775

*R*_*s*_ solution resistance, *Q*_*1*_ double layer capacitive constant phase element, *R*_*1*_ charge transfer resistance, *Q*_*2*_ coating constant phase element, *R*_*2*_ coating resistance, *R*_*p*_ polarization resistance, *n*_*1*_ and *n*_*2*_ correlation coefficient

The degradation rate of the magnesium alloy in the early stage of the implantation plays a key role in the initial surrounding tissue reaction. If the initial degradation rate is too rapid, osteolysis may occur, leading to the inferior bone tissue regeneration [43]. According to the corrosion mechanism of magnesium alloy, its corrosion degradation in physiological environment will produce hydrogen and hydroxide, resulting in the increase of pH value and the accumulation of Mg^2+^ [44]. Therefore, the degradation behaviors were investigated by measuring the pH changes and Mg^2+^ release behaviors of the different samples immersed in SBF solution. The results are shown in Fig. 4c, d. It can be seen that the blank magnesium alloy made the pH value of the solution rise rapidly, and the value arrived 9.69 at 7 days, indicating that the unmodified magnesium alloy had rapid degradation rate. The higher pH would have the damage to tissue and was not conducive to the cell adhesion and growth on the surface [45]. The corrosion resistance of Mg-OH and Mg-16 was improved, therefore their degradation rate in SBF became slower, which was confirmed by the decreased pH changes. However, the protective film formed on the surface degraded quickly with the extension of immersion time, and thus the pH value of SBF solution was still very high after 7 days of immersion (9.46 for Mg-OH and 9.36 for Mg-16, respectively). The covalent immobilization of GOCS can form a stable and dense film on the magnesium alloy surface, therefore the GOCS coating significantly reduced the effect of degradation. After loading Hep/BMP2, the pH change of SBF was further reduced, implying that the coating can effectively reduce the degradation rate of the magnesium alloy. The results of Mg^2+^ release in Fig. 4d further showed that the release amount of Mg^2+^ from the unmodified magnesium alloy was the highest, and the concentration of Mg^2+^ reached 32.67 μg/mL after 7 days. The following surface modification can gradually reduce Mg^2+^ release amount. The immobilization of GOCS and loading Hep/BMP2 can further reduce the degradation rate of the magnesium alloy in SBF, and the concentration of Mg^2+^ was only 16.82 μg/mL (Mg-GOCS) and 13.87 μg/mL (Mg-Hep/BMP2) after 7 days, respectively, indicating the decreased corrosion rate.

### Anticoagulation

**Fig. 5:**
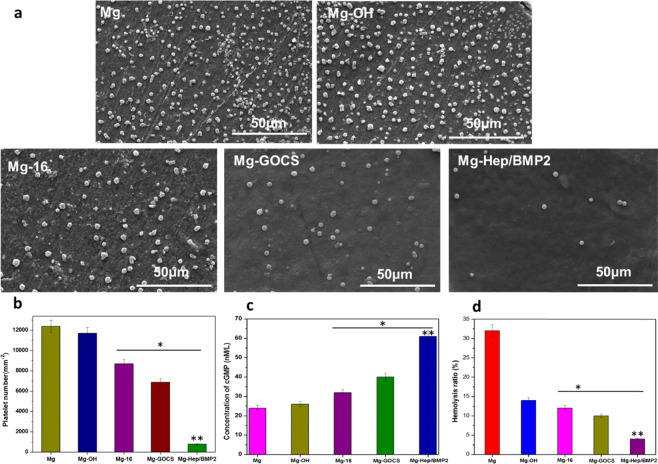
The representative SEM images (**a**) and the number (**b**) of the platelets on the different surfaces. **c**, **d** shows the expression concentration of cGMP in platelets, and the hemolysis rate of the different samples, respectively. For **b**–**d**, statistically differences are indicated by *p < 0.05 compared with the Mg and Mg-OH group, and **p < 0.05 compared with all other groups

It has been reported that the poor blood compatibility of the bone implant will cause the formation of thrombus, leading to poor blood flow at the implantation site [13], further resulting in local tissue necrosis and even implantation failure. Therefore, the anticoagulation of the different samples was investigated, and the results are shown in Fig. 5. It can be seen that a large number of platelets can be found on Mg surface (Fig. 5b), and the attached platelets displayed the state of aggregation and pseudopod spreading (Fig. 5a), implying that the platelets could have been activated. It was considered that the relative hydrophobicity of the magnesium alloy and Mg^2+^ release contributed to platelet adhesion and aggregation. The hydrophobic surface can promote fibrinogen adsorption and denaturation, which can promote platelet adhesion, aggregation, and activation [46]. At the same time, the activation of the adherent platelets may be enhanced by the released Mg^2+^, hydrogen, and high pH value [47], consequently, the adherent platelets on Mg surface expressed less cGMP (only 24 nM/L) and exhibited severely activated state (Fig. 5c). Alkali heat treatment improved the surface wettability and corrosion resistance of the magnesium alloy, which was conducive to reduce the nonspecific protein adsorption and platelet adhesion to a certain extent. However, the number of the attached platelets was still large, and the release of cGMP (26 nM/L) was enhanced slightly. The platelet number on Mg-16 decreased to 8697 platelets/mm^2^, concurrently cGMP expression was increased to 32 nM/L, indicating that it had an effective inhibitory effect on platelet adhesion and activation. On one hand, the improvement of corrosion resistance can reduce Mg^2+^ release and the change of pH value, which contributed to maintain the normal physiological environment of the blood. On the other hand, the hydrophilic carboxylic acid groups and the extended carbon chain structure were beneficial for improving the anticoagulation. Although chitosan can promote blood coagulation in some cases, the anticoagulation of the magnesium alloy was further improved after the introduction of GOCS because the number of platelet adhesion and activation was further reduced, which may be due to the fact that the excellent corrosion resistance of GOCS-modified surface can significantly reduce the stimulation and destructive effects of corrosion products on platelets. In addition, GO can also enhance the anticoagulation to some degree [20]. Heparin has excellent blood compatibility, therefore grafting or loading heparin on the surface can significantly reduce the platelet adhesion and activation [48, 49]. After loading Hep/BMP2 on Mg-GOCS, the number of platelets decreased significantly (only 796 platelets/mm^2^), moreover, no platelet aggregation was observed and the adhered platelets displayed the typical round shape. The concentration of cGMP reached 61 nM/L, indicating that the modified surface had good antiplatelet adhesion and activation ability.

In order to quantitatively detect the damage degree of the different samples to red blood cells, the hemolysis rates of the different samples were measured, and the results are shown in Fig. 5d. It can be clearly seen that the hemolysis rate of Mg was 32%, representing a serious hemolysis tendency and poor blood compatibility. Mg can be easily eroded by Cl^−^ in the physiological environment to release a large amount of Mg^2+^ and increase pH value, leading to the fusion between red blood cells and Ca^2+^ in solution [50], finally resulting in severe hemolysis phenomenon. As compared to the pristine magnesium alloy, Mg-OH and Mg-16 can effectively reduce the influences of pH value rise and Mg^2+^ release on red blood cells, leading to an obvious decrease of hemolysis rate. After the introduction of GOCS, the hemolysis rate further decreased. However, the hemolysis rate of Mg-GOCS was still higher than 5%. Heparin is a blood-friendly polysaccharide that can bind to Ca^2+^ to reduce erythrocyte damage, the hemolysis rate of Mg-Hep/BMP2 was reduced to less than 5%, which can meet the requirement of standard of ISO 10994-4:2002 and is acceptable for blood-contacting materials.

### Osteoblast growth behaviors and function expression

**Fig. 6:**
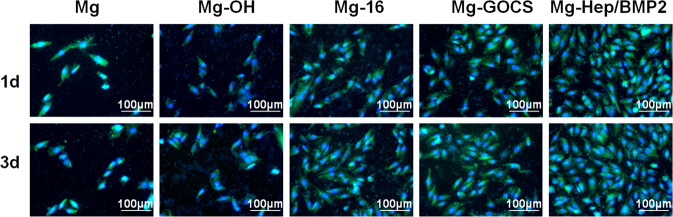
Fluorescence images of the osteoblast cells cultured on the different magnesium alloys for 1 and 3 days, respectively (green: cytoplasm; blue: nucleus) (color figure online)

Figure 6 shows the fluorescent images of the osteoblasts attached on the different surfaces. It can be seen that the number of cells attached on Mg surface was less, which may be due to the fact that a large number of hydrogen bubbles caused by rapid degradation could prevent cell adhesion [51]. At the same time, the higher pH value of the culture medium also seriously reduced the biological activities of osteoblasts. Therefore, in order to provide a good environment for cell growth, improving the corrosion resistance of magnesium alloy is an important prerequisite. Alkali heat treatment can improve the corrosion resistance to a certain extent, but the protective layer is easy to be dissolved by Cl^−^, so the number of cell adhesion on Mg-OH surface was only slightly more than that of Mg. The corrosion resistance of Mg-16 was better than Mg-OH, therefore the damage to cells was less and the number of cells on Mg-16 surface was increased. Chitosan is a kind of positively charged polysaccharide, which can interact with negatively charged osteoblasts through electrostatic force and thus promote cell adhesion and growth [52]. The graft of GOCS not only endowed the magnesium alloy surface with positively charged characteristics, but also can significantly improve its corrosion resistance, therefore more osteoblasts with favorable spreading and elongated filopodia can be observed on Mg-GOCS surface. BMP2 can promote the adhesion and proliferation of osteoblasts and promote the expression of osteogenic factor VEGF [53]. Meanwhile, as shown in Fig. 2, loading the complex of heparin and BMP2 on Mg-GOCS can create a uniform surface, therefore, Mg-Hep/BMP2 can significantly promote the osteoblast growth.

**Fig. 7:**
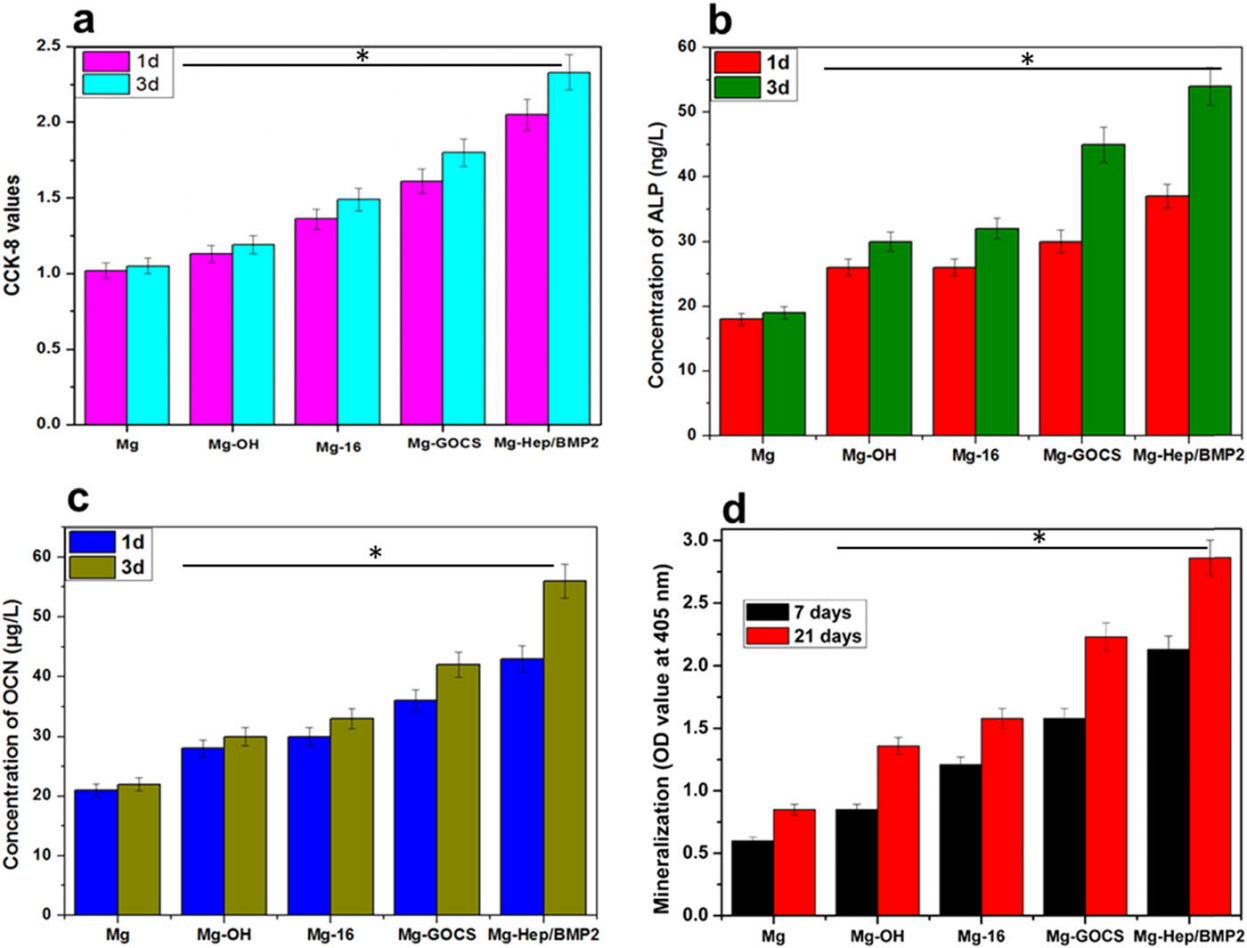
**a** CCK-8 values of osteoblast cells cultured on the different samples for 1 and 3 days, respectively. ALP (**b**), OCN (**c**) expression and mineralization (**d**) of the osteoblast cells on the different samples. Statistically differences are indicated by *p < 0.05 compared with the Mg group, and Mg-Hep/BMP2 has statistically difference (*p* < 0.05) compared other groups

The osteoblast proliferation was evaluated by CCK-8 assay, and the results are shown in Fig. 7a. The CCK-8 value of cells on the blank magnesium alloy was the lowest among all samples, moreover, there was no significant difference between 1 and 3 days, indicating that osteoblasts could hardly proliferate on the pristine magnesium surface with the extension of the culture time. Mg-OH can reduce the effect of the rapid degradation on cell behaviors and thus the CCK-8 value of Mg-OH increased slightly. The immobilization of 16-phosphonyl-hexadecanoic acid can further reduce the pH rise and Mg^2+^ release, which contributed to the adhesion and proliferation of cells, therefore the CCK- 8 value of cells on Mg-16 increased, and the value of 3 days was significantly higher than that of 1 day, suggesting that the self-assembly of 16-phosphonyl-hexadecanoic acid can promote cell proliferation. It has been confirmed that both GO and chitosan can promote osteoblast proliferation [54, 55], meanwhile, Mg-GOCS had the excellent corrosion resistance, therefore the CCK-8 value of cells on Mg-GOCS surface further increased. After loading Hep/BMP2, the enhancement of corrosion resistance can effectively avoid locally high pH values and Mg^2+^ concentrations, both of which are known to denature membrane proteins and alter membrane osmotic pressure, thereby disabling membrane channel-dependent signal transport and cell adhesion. Consequently, the CCK-8 values increased significantly for Mg-Hep/BMP2, suggesting that osteoblasts could proliferate well on the surface.

ALP is a marker of early differentiation of osteoblasts and has an important effect on osteoblast mineralization [56], therefore ALP expression in osteoblasts directly reflects the differentiation state of osteoblasts. As shown in Fig. 7b, there was no significant difference in the ALP content on the blank magnesium alloy surface when cultured for 1 and 3 days, respectively. Mg-OH and Mg-16 can reduce the effect of rapid degradation on osteoblasts, and provide a hydrophilic environment which was favorable to cell growth and differentiation, therefore the ALP content of cells increased to some degree. As shown in EIS results, Mg-GOCS not only can increase charge transfer impedance but also had a coating resistance, which can significantly retard the effects of Mg^2+^ and rapid pH rise on cell differentiation and thus promote ALP expression. At the same time, GOCS had good biocompatibility and can promote cell adhesion and proliferation, so the ALP of osteoblast on Mg-GOCS increased significantly and the value reached 45 ng/L after 3 days. BMP2 can stimulate Runx2 expression and play a pivotal role in the osteoblast differentiation [57]. After loading heparin/BMP2 on the surface, the ALP expression in osteoblasts increased significantly, and the amount was 37 ng/L for 1 day and 54 ng/L for 3 days, respectively.

OCN, also known as bone R-hydroxy glutamate protein, is mainly synthesized by osteoblasts, and some by proliferative chondrocytes, which plays an important role in regulating bone calcium metabolism [58, 59]. The detection of OCN content in cell culture medium represents one of the important methods to investigate the osteoblast functional expression. As shown in Fig. 7c, the OCN content for Mg was relatively low, and the values were 21 μg/L at 1 day and 22 μg/L at 3 days, respectively, it can be attributed to the effect of the limited cell proliferation and the Mg^2+^ release by the rapid degradation. The OCN expression of osteoblasts on Mg-OH and Mg-16 surfaces was not significantly different, but the values were significantly higher than that of the unmodified magnesium alloy, indicating that the enhancement of the corrosion resistance was beneficial to the functional expression of osteoblasts. After grafting GOCS, the expression of OCN in osteoblasts further increased due to the bioactivities of GO and chitosan. It was worth noting that OCN expression on Mg-Hep/BMP2 surface was the highest among all samples, and the value reached 56 μg/L at 3 days. BMP2 can enhance the bone formation by promoting the osteoblast differentiation from the mesenchymal stem cells and by helping in the biosynthesis of the bone matrix through control of essential factors during osteo-induction to regenerate osseous tissue [60], consequently, loading the complex of heparin and BMP2 on Mg-GOCS surface can significantly promote OCN expression.

ECM protein mineralization is the final stage of osteoblast differentiation. Figure 7d shows the mineralization behavior of osteoblasts cultured on the different surfaces for 7 and 21 days, respectively. It can be seen that all samples had a certain function of promoting cell mineralization. With the extension of culture time, better cell mineralization can be achieved. Compared with the unmodified magnesium alloy, the modified magnesium alloy had better hydrophilicity and corrosion resistance as well as good bioactivities, which was conducive to cell adhesion and growth, thus promote the mineralization of cells in varying degrees. In particular, the mineralization of cells was significantly improved after heparin and BMP2 were loaded on the surface.

## Conclusion

The complex of heparin and BMP2 was successfully loaded on the magnesium alloy surface after the immobilization of GOCS on 16-phosphonyl-hexadecanoic acid modified magnesium alloy surface, and a multifunctional bioactive coating was achieved. The surface coating can not only significantly increase the surface wettability, but also effectively enhance the corrosion resistance and reduce degradation rate in simulated physiological environment. Furthermore, the coating can effectively reduce the adhesion and activation of platelets, minimize the hemolysis rate of the magnesium alloy, and therefore significantly improve the anticoagulation. Due to the good cytocompatibility of GOCS and the osteogenic effect of BMP2, the coating can not only significantly promote the adhesion and proliferation of osteoblasts, but also obviously upregulate ALP and OCN expression and promote osteoblast mineralization. Therefore, the method of the present study provides a new strategy for surface modification of the magnesium alloy to simultaneously improve the corrosion resistance, blood compatibility, and osseointegration of magnesium alloy; it may expand the application of magnesium alloy in the bone implants.
